# Off-resonance correction for pseudo-continuous arterial spin labeling using the optimized encoding scheme

**DOI:** 10.1016/j.neuroimage.2019.05.083

**Published:** 2019-10-01

**Authors:** Eleanor S.K. Berry, Peter Jezzard, Thomas W. Okell

**Affiliations:** Wellcome Centre for Integrative Neuroimaging, FMRIB, Nuffield Department of Clinical Neurosciences, University of Oxford, Headley Way, Oxford, OX3 9DU, United Kingdom

**Keywords:** Pseudo-continuous arterial spin labeling, Off-resonance correction, B_0_ inhomogeneity, Vessel-encoding, Perfusion imaging

## Abstract

Pseudo-continuous arterial spin labeling (PCASL) MRI has become a popular tool for non-invasive perfusion imaging and angiography. However, it suffers from sensitivity to off-resonance effects within the labeling plane, which can be exacerbated at high field or in the presence of metallic implants, leading to spatially varying signal loss and cerebral blood flow underestimation. In this work we propose a prospective correction technique based on the optimized encoding scheme, which allows the rapid calculation of transverse gradient blips and RF phase modulations that best cancel phase offsets due to off-resonance at the locations of the feeding arteries within the labeling plane. This calculation is based upon a rapidly acquired single-slice fieldmap and is applicable to any number and arrangement of arteries. In addition, this approach is applicable to both conventional PCASL and a vessel-selective variant known as vessel-encoded PCASL (VEPCASL). Through simulations and experiments in healthy volunteers it was shown that in the presence of off-resonance effects a strong bias in the strength of the perfusion signal across vascular territories can be introduced, the signal-to-noise ratio (SNR) efficiency of PCASL and VEPCASL can be severely compromised (∼40% reduction in vivo), and that vessel-selective signal in VEPCASL can be incorrectly assigned. Distortion of the spatial regions placed in the label or control conditions in the presence of off-resonance effects was confirmed in phantom experiments. The application of the proposed correction restored SNR efficiency to levels present in the absence of off-resonance effects and corrected errors in the vascular territory maps derived from VEPCASL. Due to the rapid nature of the required calculations and fieldmap acquisition, this approach could be inserted into protocols with minimal effect on the total scan time.

## 1 Introduction

Arterial spin labeling (ASL) is a non-invasive magnetic resonance imaging (MRI) technique that allows measurement and quantification of cerebral perfusion. There are different approaches to ASL: pulsed, continuous and pseudo-continuous (PCASL). PCASL benefits from a higher signal-to-noise ratio (SNR) than pulsed ASL and a superior labeling efficiency to continuous ASL; as such it has become the recommended labeling approach (Alsop et al., 2015). Vessel-encoded PCASL (VEPCASL) can be used to provide information about the different vascular territories in the brain (Wong, 2007) while maintaining the same SNR efficiency as conventional PCASL (Okell et al., 2013). Such information can be useful in the study of a range of diseases, including visualizing collateral flow patterns in patients with steno-occlusive disease (Arteaga et al., 2017) or assessing blood supply to arteriovenous malformations (Okell et al., 2019).

Both PCASL and VEPCASL invert the magnetization of the blood by applying a train of radiofrequency (RF) pulses and gradients (Dai et al., 2008; Wong, 2007). The pulse train causes a flow driven pseudo-adiabatic inversion of the blood magnetization as it flows through the chosen labeling plane. The inversion is dependent on the phase accrued by the magnetization between successive RF pulses and this phase depends on the gradients applied during and between the pulses (Wong, 2007; Wong and Guo, 2012).

A PCASL scan consists of label and control cycles in which the magnetization of the blood flowing through the labeling plane is either inverted (the blood is ‘labeled’) or not inverted (‘controlled’). The subtraction of label and control cycles removes any static tissue and yields the perfusion signal. In VEPCASL, additional transverse gradient blips are applied which cause the phase accrued by the magnetization to vary across the labeling plane. This produces label and control conditions at different locations within the labeling plane in a periodic fashion, with a characteristic spatial frequency, which we refer to as the encoding function.

The presence of B_0_ inhomogeneities in the labeling plane impacts the labeling efficiency of both PCASL and VEPCASL. Inhomogeneities increase at higher field strengths, making this effect particularly problematic for ultra-high field imaging where large variations in resonance frequencies are seen along the length of brain-feeding arteries (Teeuwisse et al., 2010). Deviations in B_0_ can also occur near metallic materials, such as those found in some dental implants. Sources of off-resonance across the labeling plane introduce phase offsets that change the phase accrued at each vessel location. Consequently, the inversion efficiency of PCASL will be reduced, leading to underestimation of cerebral blood flow. The impact of these effects can differ for different arteries, also leading to bias in the perfusion signal across different vascular territories. In addition, the encoding function present during VEPCASL labeling will be shifted away from the desired label/control locations (Berry et al., 2015a; Wong and Guo, 2012), reducing SNR efficiency and leading to vessel-decoding errors. Depending on the particular subject, hardware and field strength of a given study, standard shimming may not be sufficient to correct for B_0_ inhomogeneities (Jahanian et al., 2011).

A variety of methods have been proposed to deal with the issue of phase offsets in the labeling plane during PCASL scans. The use of unbalanced PCASL can improve performance in the presence of off-resonance, but signal loss is still problematic at larger phase offsets (Zhao et al., 2017). The multi-phase PCASL (MP-PCASL) approach introduces a phase increment between each RF pulse over successive labeling cycles in equal steps from 0 to 2π radians (Jung et al., 2010). This is in contrast to the standard PCASL label/control phase pattern where there is 0 or π phase between RF pulses. The perfusion signal is determined by fitting the data acquired across all phase offsets to a model. However, compared to the performance of PCASL without any phase offsets in the labeling plane, MP-PCASL suffers from lower SNR efficiency. OptPCASL (Shin et al., 2012) performs a series of prescans to automatically determine the phase offsets that need to be accounted for at each of the vessels in the labeling plane. Additional transverse gradients and an additional RF phase term are calculated from the prescan information and applied during a standard PCASL labeling scheme to correct for both a global phase offset in the labeling plane and offsets local to each vessel. The method currently only deals with three feeding vessels and the prescans add an extra 5 min to the total scan time. A similar method (Luh et al., 2013) was developed at 7 T that likewise only enables correction for phase offsets at three vessel locations in the labeling plane. Another technique (Jahanian et al., 2011) takes phase offsets at the vessel locations from a field map of the labeling plane. An additional RF phase term and z gradient term are then calculated to best compensate for the phase offset across the entire labeling plane. The result is a compensation for a global phase offset, but within-plane offsets specific to individual vessels may not be dealt with.

Here we propose a technique to correct for phase offsets due to B_0_ inhomogeneities in the labeling plane during both PCASL and VEPCASL scans. The method is based on the Optimized Encoding Scheme (OES) (Berry et al., 2015a), which has been adapted to take into account off-resonance at an arbitrary number of vessel locations using a rapidly acquired field map of the labeling plane. Transverse gradients and RF phase increments are then calculated using the Fourier-based OES method to compensate for the offset at each vessel location. The technique is automated, compatible with any number of vessels and requires only the addition of a field map of the labeling plane to establish the (VE)PCASL protocols. This study builds on work presented in a previous conference abstract (Berry et al., 2015b).

## 2 Theory

During a PCASL labeling cycle the magnetization within all arteries experiences repeated RF pulses with the same phase, whereas during a control cycle every other RF pulse has an additional π radians of phase to prevent inversion of the magnetization whilst matching magnetization transfer effects (Dai et al., 2008). In VEPCASL, an identical pulse train is used except for the inclusion of transverse gradient ‘blips’ between the RF pulses (Wong, 2007). These vary the phase accrual of the magnetization across the blip direction, resulting in vessels at some locations being labeled and vessels at other locations being controlled at the same time. The OES automatically defines an ideal encoding scheme for a given set of vessels and calculates the necessary transverse gradient blips and RF phase modulations that generate encoding functions to best achieve this scheme (Berry et al., 2015a).

In the original OES method the vessels were assigned either a value of −1 (for a label condition) or +1 (for a control condition). In order to allow compensation for phase accrual due to off-resonance, in this work we generalize this concept by defining the encoding scheme according to the required phase to be induced at each vessel location. This allows the OES to simply calculate the optimum gradient and RF parameters that will lead to the desired label or control condition at each vessel whilst simultaneously counteracting the effects of off-resonance. The phase offsets to be counteracted are taken as the additional phase accrued by the magnetization during one RF interval in the PCASL pulse train due to the presence of B_0_ inhomogeneities. The technique is organized similarly to the original OES method and is structured as follows:

1. Construct an “image” of the vessel locations: each vessel is represented by e^iθ^, where θ is the desired phase at the vessel location, with all other locations being assigned values of zero
2. Zero-pad this “image” and take its Fourier transform
3. Weight and mask the resulting Fourier space to up-weight lower spatial frequencies and mask higher spatial frequencies
4. Find the maximum intensity in this weighted Fourier space

θ depends on the ideal encoding scheme phase, 0 (for a label condition) or π (for a control condition), with any local phase offsets, *ψ*, subtracted. The phase required at a given vessel location *r* is therefore:

(1) θ_label_(r) = 0 – ψ(r), θ_control_(r) = π – ψ(r),

The optimized encoding function, which now includes a correction for phase offsets in the labeling plane, is taken as the spatial frequency and phase at the maximum intensity point, which corresponds to the gradient and RF parameters that best match the desired phase values at the vessel locations. For further details of the RF and gradient parameter calculations, please see Appendix A. The weighting and masking of Fourier space encourages the choice of lower spatial frequencies, making the resulting encoding more robust to subject motion. It is worth noting that the optimum spatial frequency selected in this way is not equivalent to applying an additional linear shim term during the labeling period, since the desired phase only needs to be matched at specific vessel locations, rather than across the plane as a whole, leading to much greater flexibility in the design. In addition, in order to counteract the effects of off-resonance, the applied gradient blips must always be applied with the same polarity in a manner similar to the ‘unipolar’ approach of (Wong and Guo, 2012), rather than the original ‘bipolar’ approach for VEPCASL (Wong, 2007).

This process is repeated for all the cycles of the ideal encoding scheme. For conventional PCASL, this will be two cycles: one in which all the vessels are set to the label condition, and the other in which they are all set to the control condition. For VEPCASL, the ideal encoding scheme is constructed using columns from a Hadamard matrix with a size sufficient to encode all the vessels of interest (Wong, 2007). The technique currently requires the manual input of the vessel locations and phase offsets at those locations. The optimized encoding functions are then calculated without further user intervention. Calculation times are very close to the original OES method (less than 3 s) (Berry et al., 2015a).

**Fig. 1:**
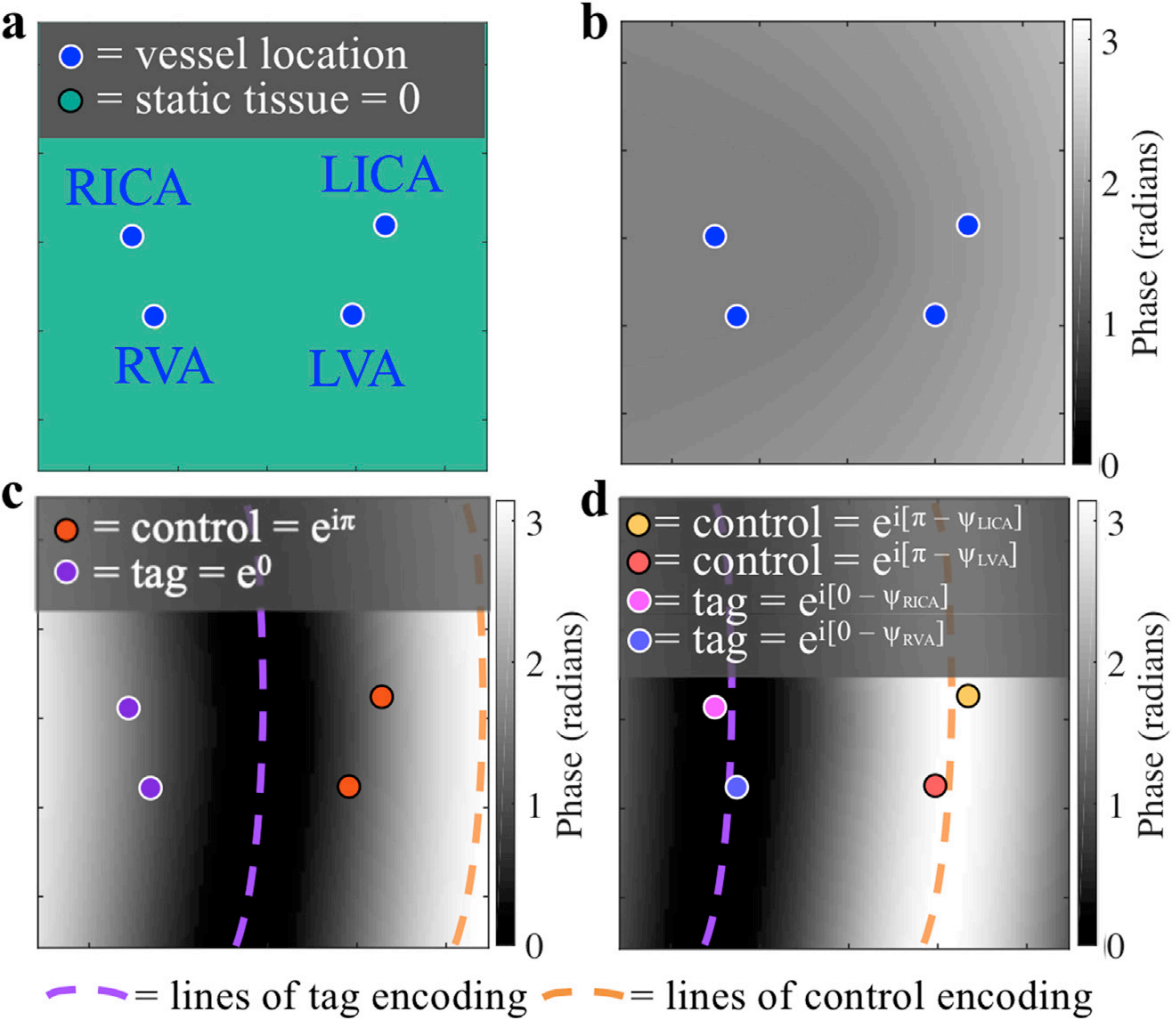
Demonstration of the proposed off-resonance correction method applied to one VEPCASL cycle. a: A group of four vessels and b: a simulated field map within the labeling plane, shown as the phase accrued during one RF interval due to off-resonance. c: In this example, the desired encoding aims to label the RICA and RVA whilst leaving the LICA and LVA in the control condition, but the combination of the prescribed transverse gradient blip with the phase accrual due to B_0_ inhomogeneities leads to a distorted and shifted encoding function, resulting in inefficient labeling. d: If the phase offsets are incorporated into the OES scheme the desired encoding is restored. Contours of zero and π phase between RF pulses, corresponding to the centre of the label (tag) and control regions, are shown with purple and orange dashed lines, respectively.

To demonstrate this process, Fig. 1 shows an example group of four vessels and a simulated field map of the labeling plane. In this case, the desired encoding involves labeling the right internal carotid artery (RICA) and right vertebral artery (RVA) whilst maintaining the LICA and LVA in the control condition. If the phase offsets due to the B_0_ inhomogeneities plotted in the field map are not taken into account then encodings chosen by the OES method will be shifted away from the intended labeling positions (Fig. 1c). However, when the phase offsets have been taken into account with the proposed method the desired encoding is restored (Fig. 1d).

The Fourier space of the “image” of complex numbers is not symmetric so, unlike the original OES method, the mask used to exclude unwanted spatial frequencies is circular, rather than semi-circular. The mask radius is calculated according to the predicted subject motion, as in the OES method (Berry et al., 2015a), so the minimum possible wavelength of an encoding function is four times the maximum predicted subject motion. Zero-padding of the Fourier space and up-weighting of lower spatial frequencies is performed as in (Berry et al., 2015a).

## 3 Materials and methods

Simulations, phantom and subject scans were all used to evaluate the OES with off-resonance correction. All scans in this study took place on a 3T TIM Verio system (Siemens Healthineers, Erlangen, Germany). All subjects were scanned under a technical development protocol approved by local ethics and institutional committees. For all calculations of the optimized encodings with a correction for off-resonance effects the zero-padded “image” matrix size was 1024 × 1024. All simulations, phantom and subject scans used a maximum predicted subject motion value of 4 mm. For VEPCASL of the four main brain-feeding arteries in the neck the ideal 8-cycle encoding matrix in (Okell et al., 2013) was used.

### 3.1 Simulations

Simulations were performed in MATLAB (MathWorks, Natick, Massachusetts, USA) to test the efficacy of the off-resonance correction in the labeling plane for both PCASL and VEPCASL. The SNR-efficiency of encodings was tested across three scenarios:

1. No phase offsets present (ψ = 0)
2. Phase offsets present, encodings uncorrected (ψ ≠ 0, but set to zero for the encoding calculations)
3. Phase offsets present, encodings corrected (ψ set to the true values)

#### 3.1.1 Generation of field offsets

**Fig. 2:**
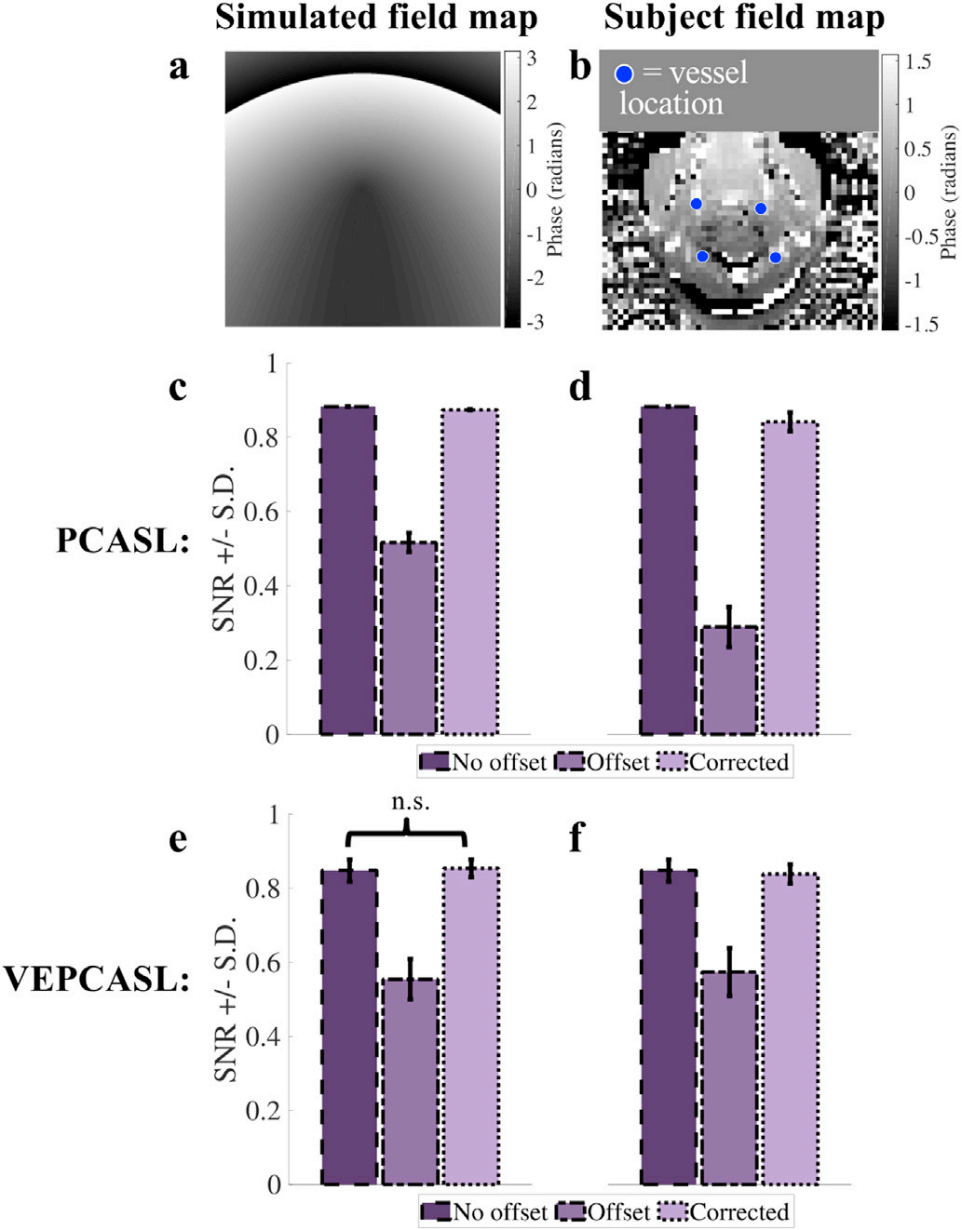
PCASL and VEPCASL four-vessel simulation results. a: Simulated and b: real field maps, with an example set of four vessels overlaid. Bar charts c – f show the mean SNR efficiency results for the PCASL (c,d) and VEPCASL (e,f) simulations across for the simulated (c,e) and real (d,f) field maps. In all scenarios the introduction of phase offsets results in a large decrease in SNR efficiency, but the application of the OES-based off-resonance correction counteracts this effect, bringing SNR efficiency back to levels present with no off-resonance effects. All SNR efficiency values within each scenario are significantly different (P < 0.05), except those marked “n.s.“. Note that when no offsets were present the PCASL inversion efficiency does not change with vessel location, and consequently the standard deviation for this scenario was always zero.

Phase offsets were taken from both simulated and acquired in vivo field maps. The simulated field maps (example given in Fig. 2a) were generated in MATLAB using a combination of a linear and quadratic field terms, giving phase offsets at the vessel locations up to approximately 1.2 radians. A real field map (Fig. 2b) was taken from a labeling plane in the neck of a healthy subject where a linear shim offset had been applied. The offsets in all the field maps were gradually increased to test the off-resonance correction against increasingly large phase offsets, up to twenty times the original values.

#### 3.1.2 Vessel arrangements in the field offsets

For both simulated and real field maps, arrangements of four vessels, representative of brain-feeding arteries in the neck, were perturbed by random distances and directions. The perturbations for each vessel were randomly chosen from a normal distribution (mean = 0 ± 2 mm) and twenty different arrangements were simulated.

#### 3.1.3 (VE)PCASL SNR-efficiency calculations

For each simulation, the longitudinal magnetization state of the blood following each encoding at each vessel location was taken from simulated magnetization curves for the PCASL/unipolar VEPCASL pulse trains, as per (Berry et al., 2015a). For PCASL, the SNR-efficiency was then computed by averaging the difference between the label and control magnetization states across vessels normalized by the ideal label-control difference (twice the equilibrium magnetization of blood). In the case of VEPCASL the SNR-efficiency was calculated according to (Wong, 2007) and averaged across all vessels.

The SNR-efficiency of the encodings of each vessel arrangement was calculated for the twenty different simulated and real field maps with increasing levels of off-resonance effects. The mean and standard deviation of the SNR-efficiency across all field maps and vessel arrangements was then computed.

### 3.2 Phantom scan protocol

In order to visualize the effects of the off-resonance correction within the labeling plane, a Eurospin test object 3 was scanned with a 12-channel head coil. This phantom contains plastic rods which could be used to represent the locations of the main brain feeding arteries in the neck. The encodings for PCASL and four vessel VEPCASL were calculated and applied. The PCASL pulse train was followed immediately by imaging of the labeling plane, as shown previously (Berry et al., 2015a). Regions placed in the label condition experience RF pulses with the same phase, leading to saturation of static spins. Regions placed in the control condition experience RF pulses with phase alternating between 0 and π, which results in a much smaller saturation effect. The label and control regions can therefore be directly visualized within the phantom as dark and light bands, respectively. For all (VE)PCASL scans the PCASL pulses used a flip angle of 20° and were Gaussian shaped with duration 600 μs, repeated every 1 ms. The gradient amplitude during the PCASL RF pulses was 6 mT/m, with negative gradient lobes applied to give a mean gradient of 0.8 mT/m, as per previous studies (Berry et al., 2015a; Okell et al., 2013). Unipolar transverse gradient blips were applied between PCASL pulses as described in (Wong and Guo, 2012) to perform vessel-encoding and/or off-resonance correction, with gradient area and associated RF phase increments as described in Appendix A.

Three scenarios were tested to match the simulations above: minimal phase offsets (standard shimming applied), phase offsets present (using modified shim settings) but not corrected, and phase offsets present but corrected for using the proposed method. Offsets were generated by manually changing the scanner X linear shim term by approximately 85 μT/m, resulting in variation in precession frequency across the phantom on the order of 500 Hz. The phase offsets needed to calculate the corrected encodings were taken from single-slice field maps acquired in less than 1 min. In the four vessel VEPCASL scans phase unwrapping of data from the acquired field map (voxel size = 4.1 × 4.1 × 5 mm^3^, TR = 400 ms, TE1 = 5.19 ms, TE2 = 7.65 ms) was required to calculate the off-resonance frequency in each voxel (via the phase difference divided by the difference in TE) before multiplying by the PCASL RF spacing (1 ms) to obtain the true phase offsets in the labeling plane. In the subsequent PCASL scans the difference between the two TEs of the field map was changed to the PCASL RF pulse spacing (1 ms) so that the phase difference between the images acquired at the two echo times corresponds to exactly the phase accrued between two PCASL pulses, avoiding the need for phase unwrapping and additional calculations (voxel size = 2.5 × 2.5 × 5.0 mm^3^, TR = 200 ms, TE1 = 5.19 ms, TE2 = 6.19 ms). Due to the smooth and high SNR nature of the resulting maps, point estimates of the phase offsets were taken from the centre of each “vessel” to pass into the OES framework.

All encodings were imaged with a spoiled-gradient echo readout similar to (Okell et al., 2010) in a single slice across the labeling plane. This allowed high spatial resolution images to be obtained which were insensitive to distortion artefacts that could arise from the applied shim offsets. The four vessel VEPCASL encodings were acquired with a labeling duration of 400 ms (voxel size = 0.9 × 0.9 × 5 mm^3^, TR = 5.92 ms, TE = 2.95 ms, acquisition window 400 ms) and the PCASL scans with a labeling duration of 300 ms (voxel size = 1.6 × 1.6 × 5 mm^3^, TR = 5.30 ms, TE = 2.64 ms, acquisition window 300 ms). The readout flip angle was 10° in both cases.

### 3.3 In vivo scan protocol

All scans were performed on healthy subjects with a 32-channel head coil. A three-dimensional multislab TOF angiography sequence was performed at the start of each scan to allow selection of a labeling plane and localization of vessels (voxel size = 0.8 × 0.8 × 1.3 mm^3^). Additionally, field maps of the labeling plane and the imaging region were acquired during each scan. The resolution of the labeling plane field map was brought into line with the TOF scan to better localize the phase offsets at the vessels (voxel size = 0.9 × 0.9 × 2.0 mm^3^, TR = 200 ms, TE1 = 5.19 ms, TE2 = 6.19 ms, acquisition time = 1 min). As per the phantom experiments, point estimates of the phase offsets were extracted from the centre of each vessel. A (VE)PCASL labeling scheme was combined with a single-shot echo-planar imaging (EPI) readout to acquire all perfusion data. The majority of the scan parameters were the same as those in (Berry et al., 2015a): tag duration = 1.4 s, voxel size = 3.4 × 3.4 × 5 mm^3^ and a single post labeling delay of 1 s. PCASL pulse train parameters were matched to those in the phantom experiments described in section 3.2 above. Background suppression consisted of a pre-saturation module and two global inversion pulses, as described in (Okell et al., 2013). The number of measurements acquired varied depending on the particular scan. A field map of the imaged slices was acquired with the same resolution as the EPI readout (voxel size = 3.4 × 3.4 × 5.0 mm^3^, TR = 200 ms, TE1 = 5.19 ms, TE2 = 7.65 ms, acquisition time = 54 s) to make distortion correction of the EPI images possible prior to analysis. T_1_-weighted structural images (voxel size = 1 × 1 × 1 mm^3^) were also acquired for use as an anatomical reference.

PCASL and VEPCASL perfusion scans were performed with the labeling plane positioned in the neck to provide perfusion data for the right and left internal carotid arteries (RICA/LICA) and the right and left vertebral arteries (RVA/LVA). The three scenarios tested were the same as those investigated with simulations (no phase offsets, uncorrected offsets and corrected offsets). Similarly to the phantom scans, to test the response of the correction to off-resonance, phase offsets were induced in the labeling plane by both shifting the automated shim adjustment volume higher in the brain and modifying the in plane and through plane shim currents. The ZX shim was shifted by approximately 350 μT/m^2^ and the Z^2^ shim by approximately 250 μT/m^2^, resulting in additional precession frequency variation across the labelling plane within the neck on the order of 250 Hz. Scans where the shim volume and gradients were set automatically by the system, which were optimized for the imaging region only, were deemed to be those with no phase offsets present since it has previously been shown that using this setup the phase offsets at the labeling plane are only 13 ± 9° (Berry et al., 2015a), giving a minimal impact on labeling efficiency. The shim settings were kept constant for all field map and perfusion data acquisitions within a given scenario.

PCASL scans of 40 measurements (acquisition time 2 min 27 s) were performed on 6 subjects (2 female, mean age 27 ± 3 years). VEPCASL scans of 80 measurements (acquisition time 4 min 45 s) were also performed in 5 of these subjects (1 female, mean age 27 ± 3.5 years). PCASL and VEPCASL scans were acquired in an interleaved fashion across the different scenarios in all of the scans, apart from one where only PCASL scans were performed. The data for one additional subject was excluded because all of the scans were severely motion corrupted.

### 3.4 In vivo image analysis

For all scenarios imaged with an induced shim offset the FMRIB Software Library tool FUGUE (Jenkinson et al., 2012) was used to correct for distortion in the EPI images. For each subject gray matter masks were created using partial volume maps, obtained with the FMRIB Software Library tool FAST (Zhang et al., 2001) from the T_1_-weighted structural images. The masks were registered to the (VE)PCASL space of each subject using a linear registration (Jenkinson, 2002) before being thresholded with a value of 0.5.

#### 3.4.1 PCASL image analysis

PCASL images were averaged across all repeats and control/label cycles were subtracted to yield perfusion images. In order to determine SNR, the mean perfusion signal within a gray matter mask was divided by the standard deviation within a background region-of-interest (ROI). The SNR was then averaged across subjects and compared for the three scenarios tested.

#### 3.4.2 VEPCASL image analysis

Images were averaged across all repeats and a Bayesian, maximum *a posteriori* (MAP) method was used to generate vessel-specific vascular territory maps (Chappell et al., 2012, 2010). For the “corrected offsets” scenario described above the MAP analysis was adapted to include shifts in the inversion efficiency curves in the labeling plane due to off-resonance. The SNR was calculated as in (Berry et al., 2015a), using signal from the dominant feeding vessel in each voxel that falls within the gray matter mask. The SNR of all vascular territories was averaged for each subject before averaging the SNR across subjects and comparing for the three scenarios tested.

### 3.5 Data availability

Raw SNR values from simulations and in vivo experiments underlying the quantitative results presented in this study are available from Mendeley Data (https://data.mendeley.com/datasets/x5p49vjsmp/draft?a=8346af34-83da-4312-b7a8-14435d2f2b90) under the CC-BY-4.0 license, in compliance with our funding bodies and institution.

## 4 Results

### 4.1 Simulations

Fig. 2 shows the results of the four vessel simulations for PCASL and VEPCASL across the field maps and scenarios tested. The mean PCASL encoding SNR-efficiency across both field maps with no offsets was 0.883. This value dropped to 0.517 ± 0.026 (simulated field map) and 0.289 ± 0.054 (real field map) when phase offsets were present but not corrected for. After correction using the adapted OES method, the mean PCASL SNR-efficiency rose back to 0.874 ± 0.001 (simulated field map) and 0.841 ± 0.025 (real field map), very close to the no offset case. A similar trend was seen for VEPCASL: the mean encoding SNR-efficiency across both field maps with no offsets was 0.848 ± 0.030, which dropped to 0.555 ± 0.055 (simulated field map) and 0.574 ± 0.065 (real field map) when offsets were present but recovered to 0.854 ± 0.024 (simulated field map) and 0.839 ± 0.026 (real field map) when these were accounted for in the OES framework. In all labeling and field map combinations every scenario was significantly different to the others according to a paired *t*-test, except for the comparison of the no offset and offset corrected scenarios for the simulated field map VEPCASL data (P = 0.095).

### 4.2 Phantom scans

**Fig. 3:**
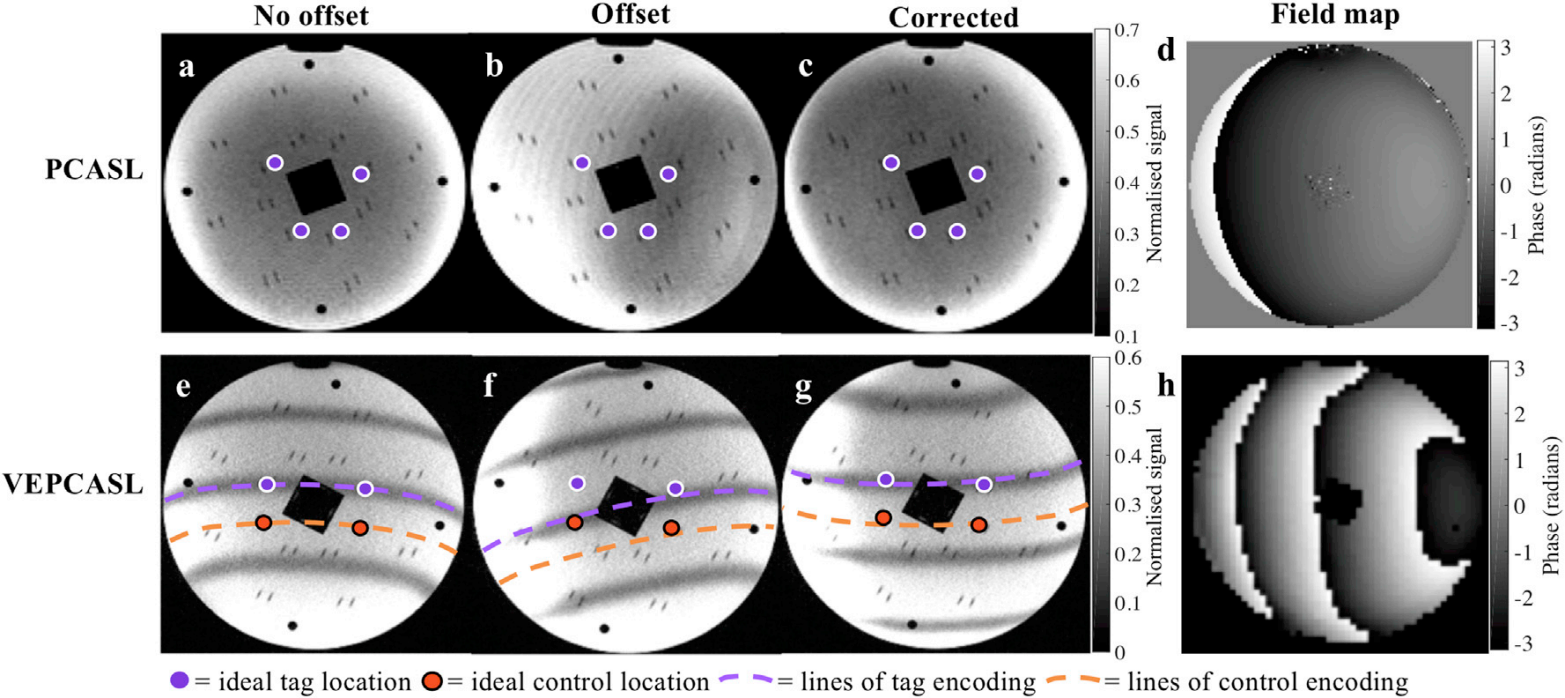
Phantom scans showing the effects of phase offsets (d,h) in the labeling plane on applied encodings (b, f) and the performance of corrected encodings (c, g) versus a scenario with no phase offsets (a,e) for both PCASL (a–d) and VEPCASL (e–h). The points in the phantom selected to represent the targeted vessels are shown with circular markers. Lower signal intensity in the phantom images corresponds to regions that will experience the label condition, and higher intensity the control condition. In the VEPCASL case, two lines of tag and control encoding, corresponding to contours of constant phase offsets between PCASL RF pulses, have been overlaid in purple and orange, respectively, to aid interpretation. The presence of off-resonance moves the labeling regions away from the targeted arteries, but the application of the off-resonance correction effectively restores the desired encoding pattern.

Fig. 3 illustrates the ability of the OES correction technique to choose encodings that account for phase offsets in the labeling plane in a phantom. The figures have been normalized to the no offset scan images in order to have the same scaling. In Fig. 3 (a) and (e) no offsets are present and the dark bands in the image, which correspond to regions that would be efficiently labeled, line up precisely with the targeted tag locations whilst avoiding those “vessels” which should be placed in a control condition. In Fig. 3 (b) and (f) the labeling regions have all been shifted away from the desired labeling locations due to the presence of off-resonance. Fig. 3 (c) and (g) show how the encodings calculated with a correction for phase offsets in the labeling plane undo the effect of off-resonance to restore the correct encoding pattern.

### 4.3 Subject scans

**Fig. 4:**
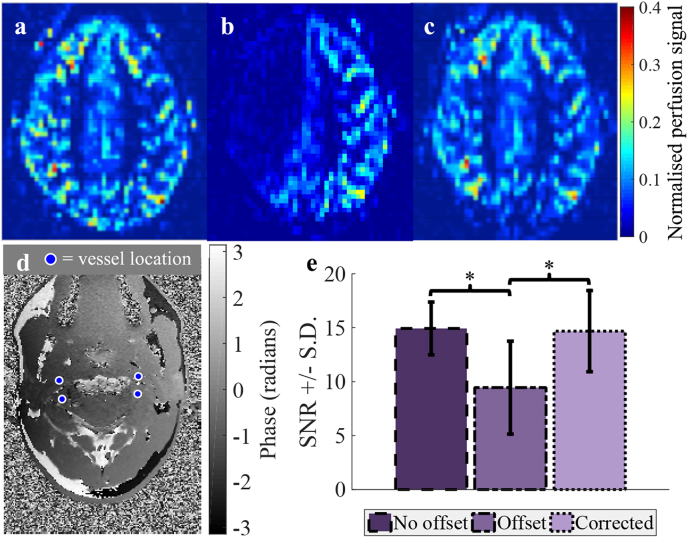
The perfusion signal in a single slice of the brain of a healthy volunteer following PCASL with a: no offsets in the labelling plane, b: offsets, uncorrected in the encoding and c: an offset corrected encoding. d: Field map of the labelling plane after shim adjustments to generate phase offsets. e: Mean SNR ±standard deviation of the perfusion signal across all subjects. *P ​< ​0.05.

Fig. 4 shows the perfusion signal in a single imaging slice of a representative subject following PCASL. The images demonstrate the non-uniform loss of signal in the brain (Fig. 4b) when there are phase offsets in the labeling plane, particularly apparent in the RICA territory in this case. If the adapted OES correction scheme is used this signal is recovered (Fig. 4c). Across all subjects the SNR in the absence of phase offsets (14.9 ± 2.4) was decreased significantly in the presence of off-resonance effects (to 9.4 ± 4.3, P = 0.003), but the proposed correction resulted in almost complete recovery of the SNR (to 14.7 ± 3.8, P = 0.003). The drop in SNR in the offset case represented a signal loss of ∼37% versus the no offset case, whereas between the no offset and corrected scenarios there was only a 2% difference (not significant, P = 0.7). Any signal loss leads to the underestimation of cerebral blood flow, which is directly related to the perfusion signal.

**Fig. 5:**
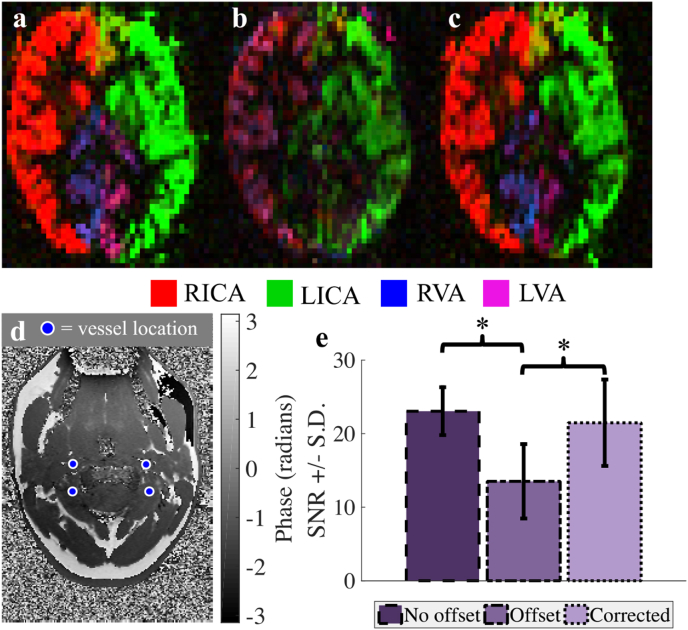
An example of vascular territories of the main brain-feeding arteries following VEPCASL. a: No offsets in the labeling plane, b: offsets, uncorrected in the encoding and c: offsets corrected in the encoding. d: Field map of the labeling plane after shim adjustments to generate phase offsets. e: Mean SNR ±standard deviation of the vessel-specific blood signal across all subjects. Signal loss and poor vessel-decoding are observed when phase offsets are present, but these are recovered through the use of the proposed method. Color represents the arterial origin of the blood signal, as shown in the legend. *P ​< ​0.05.

Fig. 5 shows vascular territory maps for a single imaging slice following VEPCASL in a different subject. When there are phase offsets in the labeling plane the distortion of the vessel-encoding functions results in signal degradation and miss-assignment of the signal to the relevant feeding arteries during the analysis (Fig. 5b). When the proposed correction scheme is used to calculate encodings which account for off-resonance effects the vascular territories are restored to their correct configuration (Fig. 5c). As in the PCASL case, the SNR calculated when no phase offsets are present (23.1 ± 3.2) is significantly degraded in the presence of off-resonance effects (to 13.5 ± 5.1, a 41% reduction, P = 0.003), but this is recovered when the proposed correction scheme is used (to 21.5 ± 5.9, P = 0.003). There was no significant difference between the no offset and corrected SNR values (P = 0.4).

## 5 Discussion

The proposed method for correcting phase offsets in the labelling plane during PCASL and VEPCASL acquisitions has been shown to work well in simulations, phantom experiments and healthy volunteer scans. The significant and non-uniform loss of signal (∼40% on average) in the perfusion maps of healthy subjects due to off-resonance effects was completely reversed using this correction technique, ensuring that SNR and CBF quantification are minimally affected by sources of off-resonance in the labelling plane. This could be particularly advantageous in cases where field inhomogeneities are severe, such as when scanning at ultra-high field, if the subject has dental implants, or if blood supply from the external carotid artery is of interest (e.g. for patients undergoing encephaloduroarteriosynangiosis), since off-resonance effects could be larger towards the edge of the neck. For VEPCASL, the presence of off-resonance effects also causes inaccuracies in the assignment of signal to the relevant brain-feeding arteries, which is also corrected for using the proposed technique. Since the calculations required are rapid (∼3 s), this approach could also be easily implemented on the scanner console.

In common with other methods that attempt to directly compensate for off-resonance effects during the acquisition (Jahanian et al., 2011; Luh et al., 2013; Shin et al., 2012), the proposed technique does not result in a loss of SNR efficiency during the ASL scan. This is in contrast to methods that acquire larger numbers of (VE)PCASL cycles and attempt correct for off-resonance in post-processing (Jung et al., 2010; Wong and Guo, 2012). The reliance on larger numbers of (VE)PCASL cycles also makes such methods less well-suited to angiographic applications based on PCASL, e.g. (Koktzoglou et al., 2011; Kopeinigg and Bammer, 2014; Lindner et al., 2015; Okell et al., 2010; Robson et al., 2010; Suzuki et al., 2018; Wu et al., 2013), where typically only a single average of each PCASL cycle is acquired to allow high spatial and temporal resolution to be obtained. Therefore, increasing the number of required (VE)PCASL cycles increases the scan time proportionally.

However, the direct correction methods do require additional pre-scans, adding to the total protocol time. The proposed method only requires the acquisition of a single-slice field map at the location of the labeling plane, which took only 1 min in this study, considerably less than the 5 min pre-scan of (Shin et al., 2012). With further refinements (such as the addition of parallel imaging), we envisage this fieldmap acquisition could be shortened considerably. In this study we used TOF images to identify the labeling plane and vessel locations, but if such information is not available, it is also possible to identify the vessel locations using the fieldmap magnitude image to prevent additional scan time being required. In our current implementation, some additional time is also required for the scan operator to manually identify the vessel locations and extract the phase offsets at these locations, but this can be performed whilst other scans are running to avoid increasing the total protocol duration and we envisage much of this could be automated in future work. An additional advantage of the proposed method is that it can correct for off-resonance effects when more than three vessels are present, unlike previous approaches (Jahanian et al., 2011; Luh et al., 2013; Shin et al., 2012). However, to ascertain the relative benefits and robustness of each technique a direct comparison would be useful as part of future work, including the application of these approaches in situations where off-resonance effects are most problematic, such as near metallic implants or at ultra-high field.

As with the other off-resonance correction methods, the proposed technique makes the assumption that throughout scanning the phase offsets remain constant. This could be violated if there is considerable B_0_ drift during the PCASL scan, or if there is significant subject motion after the acquisition of the fieldmap. The former effect could be compensated for by implementing real-time frequency adjustments, but the robustness of the proposed approach in less compliant subjects needs to be assessed prior to application in such cohorts.

Although the mechanics of the proposed method are similar to the original OES technique, the incorporation of off-resonance information results in very different encoding schemes, giving significant improvements in SNR and vessel-decoding accuracy, as shown in Fig. 5. However, in common with the original OES approach, there is an assumption that the vessels can be treated as points, rather than structures with finite width. It should be possible to account for the actual vessel cross-section size within the labeling plane using TOF data, such that all points within each vessel are assigned a value specifying the desired phase within this vessel. However, this would require more sophisticated processing, including vessel segmentation, increasing the complexity and processing time of this method. In addition, the use of the mask within the OES process to prevent the choice of high spatial frequency encoding functions should also prevent any significant variation in inversion efficiency across each vessel, making the point-like assumption a good approximation. For this study phase offsets were also extracted from the fieldmap data at a single point corresponding to the vessel centre. Due to the smooth nature of fieldmaps and minimal noise in the resulting phase difference images this was sufficient to achieve robust off-resonance corrections in phantom and in vivo experiments. However, for more rapid fieldmap acquisitions with lower SNR, increased robustness to noise could be achieved by averaging the phase offset map across the whole vessel.

Although we have only demonstrated the application of the proposed technique to four arteries within the neck, it is theoretically applicable to any number and arrangement of vessels. This could make acquisitions in which the labeling plane is positioned higher in the brain (Okell et al., 2019; Wong, 2007; Wong and Guo, 2012) more robust to off-resonance effects. Preliminary simulations (see Supplementary Fig. 1) demonstrate that the proposed method should be similarly effective when used with larger numbers of vessels. However, as mentioned above, the correction of off-resonance effects relies on the use of transverse gradient pulses of consistent polarity, equivalent to the unipolar approach for VEPCASL proposed by (Wong and Guo, 2012). Recent work has shown that such an approach results in the creation of many angled labeling planes, when considering the action of the PCASL pulses in both the through-plane and within-plane directions (Suzuki et al., 2019). The interaction of these tilted planes with the tortuous vessels above the circle of Willis is likely to be complex, undermining the assumption that we can consider only the location of the vessels within a 2D plane with the OES scheme. Even for relatively straight vessel segments, if the vessel direction deviates strongly from the normal vector of the labeling plane, the interaction with these tilted labeling planes may start to become significant. Therefore, before application in such situations, the OES method would need to be adapted to account for the 3D vessel geometry, which is likely to be complex. Alternatively, the PCASL pulse train gradient and RF parameters could be modified to allow labeling within a narrower plane, making the 2D assumptions of the OES more reliable, although the impact of such changes on inversion efficiency and the spatial encoding functions would need to be carefully assessed.

In this study, a relatively short post labeling delay of 1 s was used. This resulted in higher SNR ASL images than would be possible with the recommended post labeling delay of 1.8 s (Alsop et al., 2015), allowing scan times to be kept short so that multiple PCASL and VEPCASL scans with and without off-resonance corrections could be performed in the same scan session. However, it also limited the ability to obtain quantitative CBF estimates from these data sets (Alsop et al., 2015). Nevertheless, the improvements in SNR efficiency and robustness to off-resonance effects found with the proposed approach arise from changes in the inversion efficiency of the (VE)PCASL pulse train and therefore these benefits will be maintained for any choice of labeling duration and post labeling delay.

Finally, it is worth noting that the proposed approach aims to best match the desired phase at each vessel location, which is defined only by the ideal encoding and the phase offsets at these locations. However, since the inversion efficiency and the shape of the encoding function depend on the velocity of the blood at the labeling plane (see Supplementary Fig. 2) the resulting SNR efficiency will also vary with velocity. This should be taken into account when predicting the performance of any (VE)PCASL approach, including this correction scheme, particularly in patients with unusually high or low flow speeds at the location of the labeling plane.

## 6 Conclusions

The proposed technique effectively compensates for signal loss induced by off-resonance effects for both PCASL and VEPCASL, maximizing SNR efficiency and preventing CBF underestimation. It requires only a single-slice fieldmap acquisition of the labeling plane, the required corrections are rapidly calculated and, unlike previous approaches, the technique is applicable to more than three vessels. However, the application of this approach to labeling the tortuous vessels above the circle of Willis requires further work.

## Appendix A: Gradient and RF calculations

In this appendix we consider how to calculate the appropriate transverse gradient and RF parameters that correspond to the spatial frequency (**k**) and phase (ξ) at the maximum intensity point in weighted Fourier space. If we define **k** and ξ such that the phase induced by the transverse gradient blip, *θ*_*g*_, at location **r** is:

(A.1) *θ*_*g*_ ​= ​**k** ⋅ **r** ​+ ​ξ

Then, using the conventions of (Wong, 2007), the distance of a label location from isocentre along the transverse gradient direction, *a*, can be found by setting *θ*_*g*_ = 0:

(A.2) *a* = -ξ / |**k**|

Similarly, the distance between label and control locations, *b*, along the transverse gradient direction is found by considering that the phase difference between these locations, Δ*θ*_*g*_ = π:

(A.3) *b* = π / |**k**|

The transverse gradient blip can then be defined as having a direction aligned with **k** and area, *G*_*0*_, given by (Wong, 2007):

(A.4) *G*_*0*_ = π / γ*b* = |**k**|/γ

Where γ is the gyromagnetic ratio for hydrogen. The additional RF phase increment, *ϕ*_*i*_, for pulse *i,* required in additional to the standard PCASL phase increment, is then given (for the unipolar approach required here) by (Wong and Guo, 2012):

(A.5) *ϕ*_*i*_ = *i**π**a / b* = -*i*ξ
