# Supplementary material for: Off-resonance correction for pseudo-continuous arterial spin labeling using the optimized encoding scheme
Source: Neuroimage. 2019 Oct 1;199:304–12. doi: 10.1016/j.neuroimage.2019.05.083 (PMC6892252; doi:10.1016/j.neuroimage.2019.05.083)
Supplement: Multimedia component 1 [file mmc1.pdf]

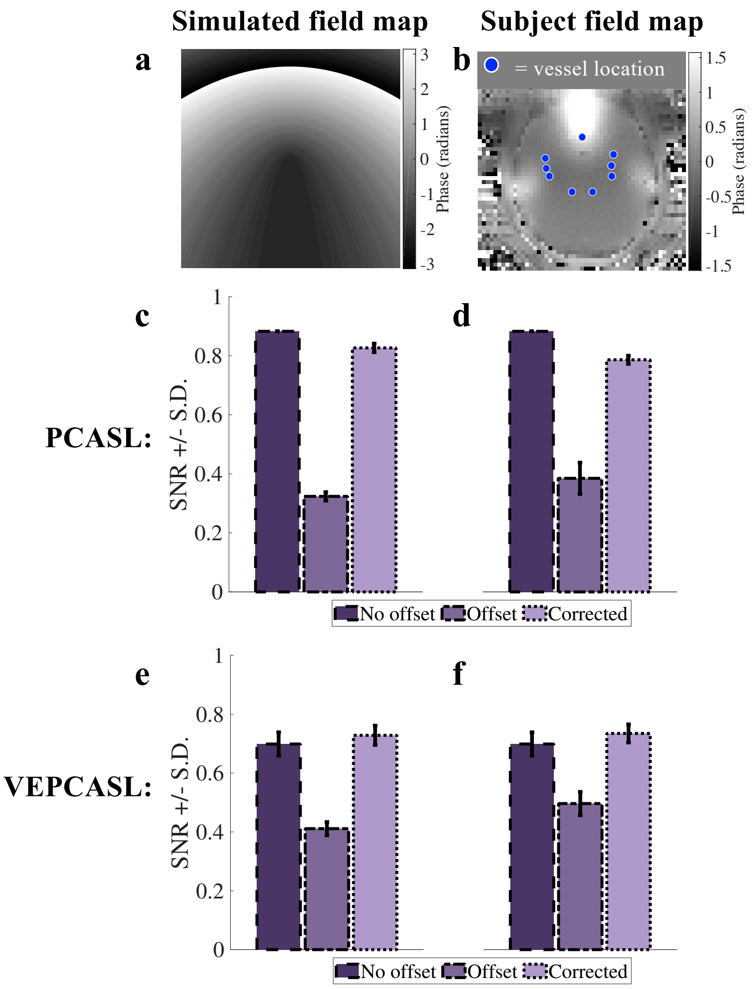

Supplementary Figure 1: Preliminary simulations of the proposed off-resonance correction scheme applied to nine vessels, representing arterial branches above the circle of Willis. Vessel locations and phase offsets were varied as per the four vessel simulations described in the methods section. a: Simulated and b: real field maps, with an example set of nine vessels overlaid. Bar charts c – f show the mean SNR efficiency results for the PCASL (c,d) and VEPCASL (e,f) simulations across for the simulated (c,e) and real (d,f) field maps. As per the four-vessel simulation results shown in Figure 2, the introduction of phase offsets results in a large decrease in SNR efficiency, but the application of the OES-based off-resonance correction counteracts this effect, bringing SNR efficiency close to levels present with no off-resonance effects. Note that VEPCASL SNR efficiency is lower for nine vessels than for four due to the increased complexity of the problem, as shown previously (Berry et al., 2015a). All SNR efficiency values within each scenario are significantly different (P < 0.05).

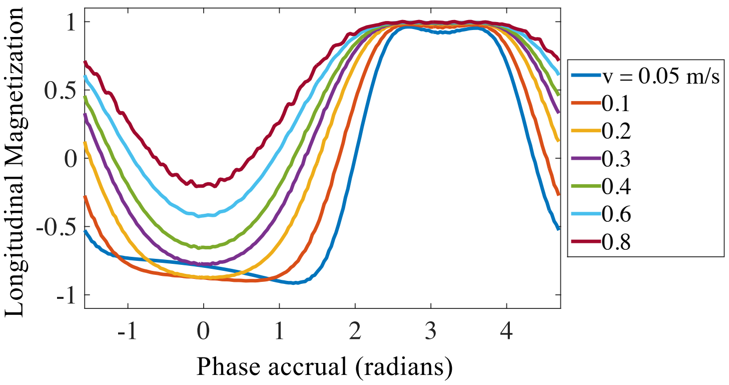

Supplementary Figure 2: Unipolar VEPCASL encoding functions derived from Bloch simulations (as described in (Berry et al., 2015a)) for a range of average flow velocities, v, assuming a laminar flow profile.
